# Litigation After Nasal Plastic Surgery

**Published:** 2011

**Authors:** Ebrahim Razmpa, Babak Saedi, Amin Safavi, Ebrahim Shahsavari, Amir Arvin Sazgar, Farzaneh Massihi, Hasan Tofighi

**Affiliations:** 1 Department of otorhinolaryngology, Tehran University of Medical Sciences, Tehran, Iran; 2 Department of otorhinolaryngology, Tehran University of Medical Sciences, Tehran, Iran; 3 Department of otorhinolaryngology, Tehran University of Medical Sciences, Tehran, Iran; 4 Department of legal medicine, Tehran University of Medical Sciences, Tehran, Iran; 5 Department of otorhinolaryngology, Tehran University of Medical Sciences, Tehran, Iran; 6 General physician, Tehran University of Medical Sciences, Tehran, Iran; 7 Department of legal medicine, Tehran University of Medical Sciences, Tehran, Iran

**Keywords:** Litigation, Malpractice, Outcomes, Plastic surgery, Rhinoplasty, Surgeons

## Abstract

**Introduction:**

Esthetic surgeries are among the commonest medical procedures in the world nowadays; and as statistics declare, there has been a rapid increase in the rate of rhinoplasty during the recent years. Hence, as the number of cosmetic surgeries rises, the increment in the number of physicians being sued is quite inevitable; either due to complication in rhinoplasties or even inability to fulfill the patients’ expectations. This article aims to clarify the aspects of causes leading to these legal claims.

**Materials and Methods:**

We designed a retrospective study according to the available files in the Iranian Organization for Forensic Medicine in which physicians were sued for the outcomes of rhinoplasty through the years 2004 to 2010. In addition, information on the patients’ demographic data, surgeons’ specialty and experience, and method of anesthesia were also collected.

**Results:**

One hundred twenty six patients entered the study among which 77 (61%) were female and 49 (39%) male. Mean age was obtained as 26.9 ± 7.7yrs. Up to 79.4% of patients had complaints concerning the cosmetic outcomes, 39.7% with respiratory and 4.8% with olfactory problems. The reason to sue the physician had a significant relationship with the patients’ age and sex, and also with the surgeons’ experience.

**Conclusion:**

There are multiple reasons impelling the patients to sue surgeons after rhinoplasty, some are related to physicians’ malpractice and some to the patients’ social and personal circumstances.

## Introduction

Surgeries for cosmetic purposes are now considered as one of the commonest medical procedures worldwide. Cosmetic surgeries keep up with the world’s development, and according to statistics, over 6 million facial plastic surgeries have been performed in the United States during the year 2007; this vast number shows an 8% increase compared to year 2006, and 460% increase compared to its previous decade (1). Although there is no precise statistics on the amount of plastic surgeries taking place in Iran, it is quite assumable that requests for plastic surgeries (rhinoplasty as a major component), have the same increasing rate as the world has.

As the number of operations rises and patients compare the works of various surgeons, and as the outcomes of rhinoplastic surgeries are supposed to satisfy the patients’ expectations and the patients desire to have a perfect nose in both cosmetics and physiological aspects. Inevitably, the surgery may not result in the patient’s desired nose, and impel the patients to sue physicians due to undesirable outcomes. Among cosmetic surgeries leading to sue a physician in developed countries, rhinoplasty accounts for about 22% of cases (2).

Considering that rhinoplasty is ranked as the fifth most common plastic surgery in these countries, in Iran it is already the commonest facial plastic surgery taking place; therefore, rhinoplasty complications are thought to be the most popular legal claims in this category.

Reasons, from which these complaints have been raised are related to diversity in anatomic structures of patients’ noses, inappropriate surgical techniques, inefficient surgeons’ experience and skills, and the unique nature of all plastic surgeries, which finally precipitates in patients’ dissatisfaction (3). On the other hand, the financial gain has encouraged non-ENT surgeons to commit such surgeries too. In this respect, we decided to investigate the possible reasons leading to suing surgeons by those patients having undergone rhinoplasty in the recent years.

## Materials and Methods

*Study Subjects*

A retrospective cross-sectional study was designed, in which one hundred twenty six case files were reviewed. The files were selected among those cases which led to sue physicians after rhinoplasty, and were referred to the Iranian Organization for Forensic Medicine in Tehran as legal claims against responsible surgeons. The study started in 2004 and ended in 2010. Selected cases included claims about complications and/or unsatisfactory outcomes after rhinoplasty; and all fulfilled the required data for this study.

*Inclusion Criteria*

Patients who had undergone rhinoplasty, were not satisfied with their surgery outcomes, and had sued their physician.

*Exclusion Criteria*

Patient files lacking information required for this study and those who had refused to sue the physician were excluded from this study.

*Ethical Approval*

Since this is a descriptive study and no intervention was made neither to case files nor to patients and physicians; ethical considerations were aimed on keeping the confidentiality of both patients’ and physicians’ personal data.

*Variables*

-Demographic data:

The age (while undergoing surgery) and sex of suing patients were obtained from their files.

-Reason leading to physician's sue:

There were thirteen district issues that complaints were raised from, they were: 1. problems with respiration, 2. problems with olfaction, 3. Cosmetics, 4. Asymmetry, 5. septal perforation, 6. ophthalmic complications, 7. Headaches, 8. Voice changes, 9. nasal discharge, 10. burns by electric cutter, 11. unilateral paresthesia, 12. problems with audition, and 13. patients’ death. Although all the patients had undertaken the surgery for cosmetic purposes, but the reason they claimed to sue the physician was not necessarily the cosmetic outcomes.

-Surgeon’s experience

The surgeon’s experience was defined as the sum of years he/she had been doing surgery till the claims due, according to this, surgeons were categorized into four groups: residents, low experienced (less than 5 years of experience), intermediately experienced (5 to 10 years of experience) and high experienced (more than 5 years of experience).

-Surgeon’s specialty

The type of residency programs which the surgeons were trained in was also taken into account; therefore they were categorized into three groups: Plastic surgeons, ENT surgeons and other specialties.

-Patient’s death

We also investigated the files to find whether rhinoplasty has precipitated in patients’ death.

-Method of anesthesia

Either of these two methods of anesthesia was used for the patients: general anesthesia or local anesthesia.

-Number of times the patient had undergone rhinoplasty

The files were investigated to know whether the first time surgery precipitated in the undesirable outcome or the following surgeries were the cause.

*Statistical Method*

Data was analyzed using Chi-Square test and T-test by applying SPSS version 11.5. The values were evaluated using descriptive statistical methods (mean ± SD) and the results were considered as significant at *P*<0.05.

## Results

Among 126 patients having entered the study, 77 (61%) were female and 49 (39%) were male. Mean age was calculated as 26.9 ± 7.7 years with the maximum of 54, and the minimum of 17 years.

Mean age for female patients was 27.3 ± 8.1 yrs and 26.4 ± 7 yrs for males; these two values did not show any significant correlations with each other (*P*=0.530).

Among female patients, the complaints were limited to aesthetic reasons for 43 (55.8%) cases, while 34 (44.2%) had complained about the functional result of their surgery. On the other hand, male patients’ complaints were limited to aesthetic reasons in only 18 (36.7%) cases and those complaints related to the functional outcomes included 31 (63.3%) cases; The difference in these values was statistically significant (*P*=0.036). Mean age for those with complaints limited to aesthetic reasons was 25.8 ± 6.5yrs, while the same value for those with complaints limited to functional outcomes was 28.11 ± 8.6 yrs; these values were not statistically significant (*P*=0.094).

**Table 1:** Sexual distribution of the claimed reasons after rhinoplasty

	Claims limited to aesthetic reasons	Claims related to functional outcomes	Total
Female	43 (55.8%)	34 (44.2%)	77 (100%)
Male	18 (36.7%)	31 (63.3%)	49 (100%)
Total	61 (48.4%)	65 (51.6%)	126 (100%)
			

According to patients’ complaints, cases were categorized into two groups: those with cosmetic reasons and those with non-cosmetic reasons; thus, 61 (48.4%) patient complaints were limited to cosmetic outcomes, while in 65 (51.6%) cases non-cosmetic reasons led to suing the physician. Details are outlined in the following table.

**Table 2:** Reason for litigation after rhinoplasty

**Reason for sue**	**Amount**	**Percent**
Aesthetic reasons	61	48.4
Aesthetic and respiratory problems	39	31
Respiratory problems	11	8.7
Olfactory problems	6	4.8
Patient’s death	2	1.6
changes in patient's voice	2	1.6
Nasal discharge	1	0.8
Burns by electric cutter	1	0.8
Unilateral paresthesia	1	0.8
Problems with audition and headache	1	0.8
Revision surgery	1	0.8
Total	126	100

The type of specialty in which the surgeon was trained, was also investigated. In 101 (80.2%) cases ENT surgeons were responsible, while there were 14 (11.1%) cases for plastic surgeons, 8 (6.3%) cases for general surgeons and 3 (2.4%) cases for maxillofacial surgeons. Data shows that percentage of complaints solely limited cosmetics among those patients who referred to an ENT surgeon counted for up to 51.5% (52 patients), while the same value for plastic surgeons was 28.6% (4 patients), 37.5% (3 patients) for general surgeons, and 66.7% (2 patients) for maxillofacial surgeons. These values were not statistically correlated (*P*=0.338).

67 (52.2%) claims were against surgeons with over 10 years of experience while 30 (23.8%) were against those with 5 to 10 years of experience and 28 (22.2) against those with less than 5 years of experience.

**Fig 1:**
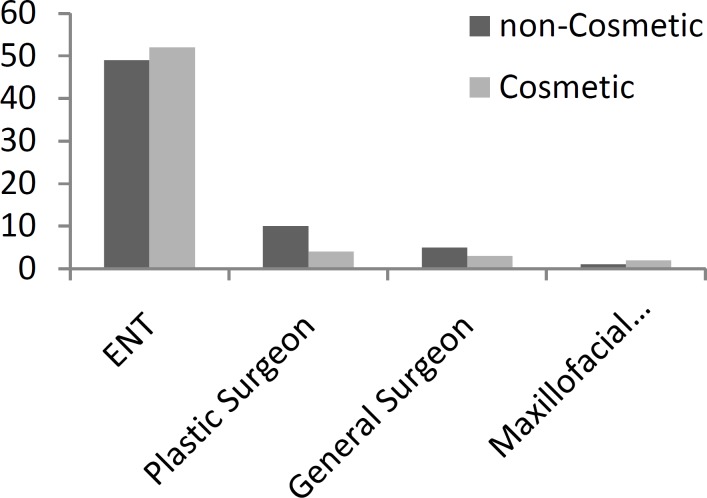
Specialty distribution of litigation files

A resident was also sued. Among surgeons with less than 5 years of experience (including the resident), claims concerning cosmetic results were more than functional outcomes; that is while among surgeons with over 5 years of experience (intermediate and high experienced surgeons) complaints were mostly due to functional outcomes (*P*=0.036). The following table outlines details on this issue.

**Table 3:** Surgeons’ experience distribution according to cause of litigation

Reason to sue	Reasons limited to cosmetics	Functional reasons	Total (%)
Surgeon’s experience			
Residents and surgeons with <5 years of experience	20 (69%)	9 (31%)	29 (100%)
5-10 years of experience	12 (40%)	18 (60%)	30 (100%)
>10 years of experience	29 (43.3%)	38 (56.7%)	67 (100%)
Total (100%)	61 (48.4%)	65 (51.6%)	126 (100%)
			

101 (80.2%) patients had undergone general anesthesia, and the remaining 25 cases (19.8%) had received local anesthesia. Among patients with general anesthesia 46.5% (47 patients) had complaints limited to cosmetics and the other 53.5% (54 patients) had complained about the functional outcomes; that’s while among patients with local anesthesia 56% (14 patients) had complaints related to cosmetics and 44% (11 patients) of the functional outcomes. The method of anesthesia and type of complaints were not statistically correlated (*P=*0.397).

In 89 (70.6%) cases, first time surgery, and in 37 (29.4%) cases, revisions had led to patients complaints. Among first timers 45% (40 patients) of complaints were limited only to cosmetics and the remaining 55% (49 patients) were due to the functional outcomes; while among those who undergone revisions, 56.8% of complaints were related to cosmetic results and the other 43.2% (16 patients) were about the functional outcomes. These values were not statistically correlated (*P*=0.227).

## Discussion

Much attention must be paid to this point that patients who request to undergo rhinoplasty are already inconvenient with their natural noses, or in some cases they may be obsessive about the perfection of their appearance; and as a matter of fact the cosmetic outcome is the thing they really look for. Although this point cannot be generalized to all patients, this study has shown that the cosmetic outcome is the main reason leading to sue in physicians; in details we can say that cosmetic outcomes has been one of the reasons to sue (along with other causes) in 79.4% of patients, and the only cause to sue in 48.4% of patients (4-6). Reasons placed in the next ranks are: problems with respiration in 39.7% (accompanied by other causes) and in 8.7% as the only cause; problems with olfaction in 4.8%; and all other causes together in 7.2% of patients. These findings suggest that physicians should evaluate the social, psychological and economic condition of patients, and also factors influencing their satisfaction prior to rhinoplasty. In this study, female suing patients were more than males; this might be because female patients are more prone to sue, but this data is not of value, since we do not know the total number of surgeries in each sex and true sex ratio of all patients undergone rhinoplasty.

Mean age of patients at surgery date was about 27 years; although most of them were aged 25 to 30, a remarkable number of them were aged over 35. According to data obtained from this study, those patients whose complaints were limited only to cosmetics had a lower mean age, meaning that younger patients are more concerned about the cosmetic outcomes while older patients would rather focus on functional outcomes along with cosmetics.

The mean time interval between surgery and date of suing was about 2 years and 4 months; this fact means that major complaints have been raised after a couple of years from the surgery date, and thus, time passage does not warrant that the patients will not sue.

Majority of sued physicians in this study had over 10 years of experience, but since we do not have any applicable data about the total number of sued and non-sued surgeons, we cannot comment on the possible relation between the surgeons experience and the reason to sue. The same issue exists about the type of surgeon’s specialty. Lower experienced surgeons were more sued due to cosmetic outcomes, while for those with more experience, there were more claims on the functional and non-cosmetic outcomes. Although existence of a claim against a surgeon never means the physician has committed malpractice, more experienced surgeons may be better at informing patients about the possible outcomes, or on the other hand, patients may relate the outcomes of a younger physician's work to his lower level of experience.

There was no significant relationship between the patients’ age and sex; mean age of both sexes were almost the same, but cosmetics was the main issue in the female group while males were mostly concerned about functional and non-cosmetic issues.

Method of anesthesia, number of times the patient had undergone surgery and the surgeon’s specialty were not significantly related to the reasons to sue. Also, because of the fact that the total number of surgeries by different specialties is unknown, the ratios of litigation between different surgeons are ambiguous. Since nasal plastic surgery is a highly litigated area of otolaryngology, therefore precise patient selection and consulting are greatly recommended before surgery planning (7-9).

## Conclusion

What this study suggests is that the process which leads suing a surgeon by the patient is a multi factorial issue; therefore some cases are caused by physicians’ malpractice, and some are dependent on the patients’ personal and social characteristics. The thing that seems necessary for every physician is to describe all medical, personal and social aspects of esthetic surgeries beside the possible complications that may cause irreversible damage to his/her face integrity. Despite the fact that almost every physician does so, the attitude of the patient to this kind of surgery should be evaluated, because he/she must be fully convinced that rhinoplasty is never meant to fulfill all his/her ideals for a perfect nose.

## References

[1] American Society of Aesthetic plastic surgery reports Nearly 11.7 million cosmetic surgical and non-surgical procedures in the United States in 2007.

[2] http://www.phudson.com/backgroudn/ligation.html.

[3] Kim DW, Lopez MA, Toriumi DM, Cummings CW, Flint PW, Harker LA, Haughey BH, Robbins KT (2009). Revision rhinoplasty. Cummings otolaryngology head and neck surgery.

[4] Mir-Akbari (2003). A survey on claims for malpractice in surgeries of nose referred to organization for Forensic medicine through years 1994 to 2000. Scientific journal of Forensic medicine.

[5] Konstantindis I, Triaridis A (2005). Long term results following nasal septal surgery focus on patient satisfaction. Auris Nasal Larynx.

[6] Shemshadi H, Azimian M, Onsori AM, Farahani MA (2008). Olfactory function following open rhinoplasty: A 6-month follow-up study. BMC Ear Nose Throat Disord.

[7] Becker S, Duncavage J (2010). Malpractice claims in nasal and sinus surgery. Otolaryngol Clin N AM.

[8] Patel M, Still T, Vaughan W (2010). Medico legal issues in endoscopic sinus surgery. Otolaryngol Clin N AM.

[9] Naeimi M, Abdali N (2009). Medical journal of Ahwaz Jondishapur University.

